# Vanadium, cobalt, zinc, and rubidium are associated with markers of inflammation and oxidative stress in a Greek population with obesity

**DOI:** 10.3389/fendo.2023.1265310

**Published:** 2023-11-22

**Authors:** Charalampia Amerikanou, Stamatia-Angeliki Kleftaki, Sotirios Karavoltsos, Dimitra Tagkouli, Aikaterini Sakellari, Evdokia Valsamidou, Aristea Gioxari, Nick Kalogeropoulos, Andriana C. Kaliora

**Affiliations:** ^1^ Department of Nutrition and Dietetics, School of Health Science and Education, Harokopio University of Athens, Athens, Greece; ^2^ Department of Chemistry, National and Kapodistrian University of Athens, Athens, Greece; ^3^ Department of Nutritional Science and Dietetics, School of Health Science, University of the Peloponnese, Kalamata, Greece

**Keywords:** trace elements, metals, obesity, oxidative stress, inflammation

## Abstract

**Introduction:**

The prevalence of obesity is rising globally, with negative effects on the socioeconomic system. As a result of its drivers which include low-grade chronic inflammation, oxidative stress, and fatty acid metabolism, this phenotype develops metabolic anomalies that exacerbate its pathogenesis. It has been discovered that metals and metalloids have substantial effects on both the immune system and metabolism and are influenced by factors connected to obesity. Although there is a known connection between metals, obesity, and related metabolic disorders, it is still under research.

**Methods:**

We determined the plasma levels of 16 metals and metalloids in 76 individuals with obesity and investigated the relationships with inflammatory and oxidative stress biomarkers in order to clarify the processes by which metals/metalloids exhibit their effects.

**Results:**

After adjusting for age, gender, BMI, physical activity level, smoking, the existence of metabolic abnormalities, and dietary intake of the corresponding metal, regression analysis revealed the following statistically significant associations; vanadium was negatively associated with oxLDL (Beta ± SE= -0.014 ± 0.005, p=0.007), zinc was negatively associated with leptin (Beta ± SE= -12.390 ± 5.226, p=0.025), cobalt was associated negatively with adiponectin (Beta ± SE= -0.030 ± 0.012, p=0.001) and positively with MPO (Beta ± SE= 0.002 ± 0.001, p=0.023), and rubidium was negatively associated with oxLDL (Beta ± SE= -1.139 ± 0.411, p=0.008) and positively with MPO (Beta ± SE= 0.324 ± 0.102, p=0.003).

**Discussion:**

The aforementioned associations highlight the need for further research, demonstrating the importance of inflammation and oxidative stress in the association between metals/metalloids and obesity-related metabolic abnormalities.

## Introduction

Obesity has reached pandemic proportions worldwide and although its “twin” pandemic, COVID-19 seems to decline, obesity and its related comorbidities still rise (1). According to the World Health Organization (WHO), obesity has nearly tripled since 1975. More than 650 million adults were obese in 2016, while overweight and obesity killed more people than underweight did in most countries (2). At the same time, obesity is associated with several metabolic abnormalities hallmarks of the metabolic syndrome such as insulin resistance, hypertension, and dyslipidemia.

Obesity has been linked to a number of pathophysiological mechanisms. The latest research supports the fact that food quality, lifestyle, genetic and epigenetic background, gut dysbiosis, and environmental and microenvironmental factors play significant roles in obesity development and progression (3). Several epidemiological and experimental studies have shown that exposure to metals may exert an “adipotropic” effect, and most heavy metals have been associated with anthropometric and metabolic parameters of obesity (4). Furthermore, trace elements regulate several biological processes that underlie the development and progression of obesity and metabolic abnormalities (5).

Obesity is considered a low-grade systemic inflammatory condition where increased infiltration of proinflammatory cytokines and macrophages into the adipose tissue is evident, also contributing to the development of metabolic disorders (6). Additionally, systemic oxidative stress is induced in obesity through several processes, i.e., oxidative phosphorylation, superoxide generation, and protein kinase C activation, and is associated with body mass index (BMI), visceral fat, and metabolic syndrome (7, 8). Interestingly, metals are linked with the immune system and mineral deficiencies impair immune function, whereas inflammation and oxidative stress modulate the metabolism and bioavailability of trace elements (5, 9). On the contrary, exposure to metals affects mitochondrial mechanisms, disrupts the endocrine system, and induces chronic inflammation and oxidative stress, contributing to obesity and metabolic disorders (10).

The relationship between trace elements’ levels and obesity and metabolic risk, as well as the associations of metals with anthropometric indices, have been well documented in a number of case-control studies (4, 11–13). Specifically, these data process the relationship between metals and the phenotypes associated with the disease, without an in-depth exploration of the mechanisms involved in the above. Therefore, studies that explore the potential link between metals/metalloids, inflammation, and oxidative stress in obesity are lacking. Therefore, the aim of the present work was to examine the associations between plasma metal and metalloid levels and inflammatory and oxidative stress biomarkers in metabolically unhealthy people with obesity in Greece.

## Methods

### Study design and participants

Hereby, 76 metabolically unhealthy individuals with central obesity from the NCT04785573 (clinicaltrials.gov identifier) study were included. Additional details about this cohort are provided in Gioxari et al. (14). More specifically, men and women above the age of 18, with central obesity defined as waist circumference (WC) above 94cm in men and above 80cm in women, and at least one metabolic abnormality expressed as triglycerides (TG) level ≥150 mg/dL, or high-density lipoprotein (HDL) cholesterol ≤40 mg/dL in men and ≤50 mg/dL in women, or increased blood pressure ≥130/85 mm Hg, or elevated fasting blood glucose ≥100 mg/dL were included. Pregnancy, lactation, untreated thyroid disease, use of any supplement within 3 months before recruitment, drug or alcohol abuse, and psychiatric or mental disorders constituted exclusion criteria. Stable body weight for at least 3 months before the enrollment and a moderately active lifestyle were additionally required.

The Harokopio University Ethics Committee (ID protocol: 1799/13-06-2019) approved the protocol of this study which was conducted in accordance with the principles contained in the latest updates of the Declaration of Helsinki and the Data Protection Act. All participants provided written informed consent to take part in this study which took place in Athens, Greece, between 2021 and 2022.

### Medical, demographic, anthropometric, biochemical, and lifestyle variables

Epidemiological information such as demographics (age, sex, and marital status), lifestyle characteristics (smoking, physical activity, and quality of life), and medical history were collected. Qualified dietitians measured anthropometric indices (weight, height, waist circumference (WC), and body mass index (BMI) using weight in kg divided by the square of height in meters (m^2^)). Finally, 24h recalls were collected for the extraction of metal dietary intake with the application of Nutritionist Pro™ software (Axxya Systems, Stafford, TX, USA).

Fasting blood (20mL) was drawn for serum and plasma isolation. Serum was used for biochemical measurements of glucose, lipids, and hepatic function with an automatic biochemical analyzer (Cobas 8000 analyzer, Roche Diagnostics GmbH, Mannheim, Germany).

### Inflammatory and oxidative stress biomarkers assessment

Isolated serum was also used for the measurement of C-reactive protein (CRP) in an automatic biochemical analyzer (Cobas 8000 analyzer, Roche Diagnostics GmbH, Mannheim, Germany). Interleukin-6 (IL-6), tumor necrosis factor-a (TNF-a), adiponectin, leptin (R&D Systems, Inc., Minneapolis, MN, USA), oxidized low-density lipoprotein (oxLDL) (Mercodia, AB, Uppsala, Sweden), and myeloperoxidase (MPO) (Thermo Fisher Scientific Inc., Waltham, MA, USA) were quantified with sandwich enzyme-linked immunosorbent assay (ELISA). All measurements were performed in duplicates.

### Quantification of metals/metalloids in plasma

Plastics contacting blood were properly cleaned and soaked in diluted HNO_3_ (Merck, Darmstadt, Germany) prior to being rinsed with ultrapure water of 18.2 MΩ cm (Millipore, Bedford, MA, USA). Samples dilution was performed with micropipettes that had undergone routine calibration. Class A volumetric glassware was used for the preparation of all required solutions. A mixture of HNO_3_ (suprapur 65%) and H_2_O_2_ (suprapur 30%) (Merck) was used for samples’ digestion, following a slightly modified version of the process described by Jin et al. (15) and Batáriová et al. (16), according to that described in Amerikanou et al. (17). A single collision cell mode ICP-MS (Thermo Scientific ICAP Qc, Waltham, MA, USA) was used for digested samples’, and pure He was used for kinetic energy discrimination (KED). For the correction of matrix-induced signal suppressions and instrument drift, internal standards (45Sc, 103Rh) were used. The average of two measurements for each sample was used to process the data statistically. According to US EPA (18), the limits of detection (LOD) ranged from 0.03 µg L^-1^ for Cs and Tl to 0.8 for Fe. Values below the LOD were given according to the method detection limit divided by √2.

### Statistical analysis

Continuous variables are presented as mean ± standard deviation (SD) or median (interquartile range, IQR) according to their distribution, and qualitative variables as counts (%). Student’s t-test and Mann-Whitney U test were used for the comparisons of means between two independent groups. Correlations analysis of metals with inflammatory/oxidative stress biomarkers was performed with the Pearson correlation test or with Spearman’s rank correlation test depending on the distribution. To test the associations displaying a significant bivariate correlation, multivariate linear regression analyses were carried out. Statistical analysis was performed using SPSS 21.0 (IBM, SPSS Inc., Chicago, IL, USA), and p-values below 0.05 were considered significant.

## Results

The basic characteristics of the participants are shown in **Table 1**. The average age was 54.2 ± 11.7 years, BMI 32.0 ± 4.5 kg/m^2^, and the majority were women (65.8%), non-smokers (77.6%), and married (76.3%). Hypertension (73.7%) and hyperlipidemia (89.5%) were the most frequent metabolic abnormalities. Plasma metal concentrations are presented in **Table 2**.

**Table 1:** Baseline characteristics of the study population.

Variables	N=76
Age (years), mean ± SD	54.2 ± 11.7
Sex (male/female), N (%)	26 (34.2)/50 (65.8)
BMI (kg/m^2^), mean ± SD	32.0 ± 4.5
WC (cm), mean ± SD	104.6 ± 10.3
SBP (mm/Hg)	140.5 ± 12.0
DBP (mm/Hg)	75.6 ± 9.7
Marital status, N (%)	
Married	58 (76.3)
Divorced	2 (2.6)
Single	10 (13.1)
In a relationship	3 (4.0)
Widowed	3 (4.0)
Metabolic abnormality, N (%)	
Hypertension	56 (73.7)
Hyperglycemia	23 (30.3)
Hyperlipidemia	68 (89.5)
Lifestyle	
Smoking, N (%)	17 (22.4)
PAL (total MET- min/week), median (IQR)	715.5 (1470.8)
AIS, median (IQR)	5.0 (6.0)
CESD-R, mean ± SD	12.9 ± 10.0
RSES mean ± SD	32.9 ± 5.2
PCS-12, mean ± SD	46.6 ± 10.2
MCS-12, mean ± SD	47.7 ± 10.8
Biochemical parameters	
Glucose (mg/dl), median (IQR)	97.0 (14.0)
TC (mg/dl), mean ± SD	185.8 (27.1)
TG (mg/dl), mean ± SD	118.6 (41.7)
HDL (mg/dl), mean ± SD	52.0 (10.3)
LDL (mg/dl), mean ± SD	112.5 ± 22.0
SGOT (iu/l), mean ± SD	15.6 ± 4.3
SGPT (iu/l), mean ± SD	17.8 ± 7.6
γ-GT (iu/l), mean ± SD	18.6 ± 8.0
ALP (U/L), mean ± SD	65.9 ± 17.5
Inflammatory/oxidative stress biomarkers	
CRP (mg/L), mean ± SD	3.0 ± 2.6
IL-6 (pg/mL), mean ± SD	2.7 ± 1.6
TNF-α (pg/mL), mean ± SD	1.0 ± 0.3
Leptin (ng/mL), median (IQR)	35.7 (52.2)
Adiponectin (μg/mL), mean ± SD	11.8 ± 7.7
MPO (ng/mL), mean ± SD	82.7 ± 58.5
oxLDL (U/l), mean ± SD	73.3 ± 14.2

Continuous variables are presented as mean ± SD or median (IQR) according to their distribution and qualitative variables as counts (%). BMI, body mass index; WC, waist circumference; SBP, systolic blood pressure; DBP, diastolic blood pressure; PAL, physical activity level as assessed by International Physical Activity Questionnaire Short Form; AIS, Athens Insomnia Scale; CESD-R, Center for Epidemiologic Studies Depression Scale Revised; RSES, Rosenberg Self-Esteem scale; PCS-12, Physical Component Score; MCS-12, Mental Composite Score; TC, total cholesterol; TG, triglycerides; HDL, high-density lipoprotein; LDL, low-density lipoprotein; SGOT, serum glutamic-oxaloacetic transaminase; SGPT, serum glutamic-pyruvic transaminase; γ-GT, γ-glutamyl transferase; ALP, alkaline phosphatase; CRP, C-reactive protein; IL-6, interleukin-6; TNF-α, tumor necrosis factor-α; MPO, myeloperoxidase; oxLDL, oxidized low-density lipoprotein.

**Table 2:** Plasma metals and metalloids of the study population.

Metals	N=76 Plasma levels (μg/L)
V	0.83 ± 0.50
Cr	6.43 ± 4.57
Mn	3.79 ± 4.32
Fe	1058 ± 367.6
Co	0.62 ± 0.54
Ni	4.16 ± 2.50
Cu	1158 ± 154.1
Zn	763 ± 538
As	0.60 ± 0.51
Se	48.7 ± 29.5
Rb	335 ± 63.1
Sr	29.4 ± 11.1
Cd	0.20 (0.29)
Cs	0.77 ± 0.33
Ba	2.46 (4.67)
Tl	0.02 (0.11)

Plasma metal levels are presented as mean ± SD or median (IQR) for Cd, Ba, and Tl. V, Vanadium; Cr, Chromium; Mn, Manganese; Fe, Iron; Co, Cobalt; Ni, Nickel; Cu, Copper; Zn, Zinc; As, Arsenic; Se, Selenium; Rb, Rubidium; Sr, Strontium; Cd, Cadmium; Cs, Caesium; Ba, Barium; Tl, Thallium.

**Table 3** shows some intriguing correlations between plasma metals/metalloids and inflammatory/oxidative stress biomarkers. More specifically, CRP correlated positively with Cu (Rho= 0.357, p=0.002), whereas IL-6 negatively with Rb (Rho= -0.319, p=0.014). TNF-a correlated positively with Se (Rho= 0.248, p=0.048) and negatively with Cu (Rho= -0.302, p=0.012). Leptin showed a positive correlation with Cu (Rho= 0.280, p=0.017), whereas a negative one with Fe (Rho= -0.274, p=0.021), Ni (Rho= -0.238, p=0.045), and Zn (Rho= -0.334, p=0.004). Adiponectin had an inverse correlation with Co (Rho= -0.276, p=0.037) and Ni (Rho= -0.285, p=0.030), and a positive one with Se (Rho= 0.323, p=0.016). Regarding MPO, positive correlations with Co (Rho= 0.321, p=0.012), Cu (Rho= 0.264, p=0.037), and Rb (Rho= 0.445, p<0.01) were observed. Finally, oxLDL negatively correlated with vanadium (V) (Rho= -0.285, p=0.018) and Rb (Rho= -0.312, p=0.009).

**Table 3:** Correlation analysis between plasma metals/metalloids and inflammatory/oxidative stress biomarkers.

		V	Cr	Mn	Fe	Co	Ni	Cu	Zn	As	Se	Rb	Sr	Cd	Cs	Ba	Tl
**CRP (mg/L)**	**Correlation Coefficient**	-0,164	0,017	-0,134	-0,219	-0,070	-0,198	**0,357**	-0,193	-0,073	0,057	0,105	-0,084	0,076	0,064	0,060	-0,115
	***P***	0,177	0,887	0,266	0,064	0,563	0,095	**0,002**	0,104	0,544	0,642	0,382	0,487	0,530	0,597	0,614	0,335
**IL-6 (pg/mL)**	**Correlation Coefficient**	-0,256	0,054	-0,220	-0,228	-0,038	-0,059	-0,011	-0,215	0,174	0,134	**-0,319**	0,126	0,246	-0,146	-0,156	-0,052
	***P***	0,052	0,687	0,086	0,075	0,777	0,650	0,932	0,100	0,183	0,322	**0,014**	0,347	0,060	0,265	0,230	0,692
**TNF-α (pg/mL)**	**Correlation Coefficient**	-0,130	-0,121	-0,077	0,129	-0,103	-0,069	**-0,302**	-0,002	0,126	**0,248**	-0,110	0,125	0,127	0,034	0,031	-0,054
	***P***	0,305	0,338	0,533	0,299	0,412	0,579	**0,012**	0,986	0,308	**0,048**	0,374	0,321	0,308	0,784	0,801	0,663
**Leptin (ng/mL)**	**Correlation Coefficient**	-0,209	0,060	-0,089	**-0,274**	-0,029	**-0,238**	**0,280**	**-0,334**	-0,153	0,006	-0,143	-0,032	0,068	-0,212	-0,231	-0,178
	***P***	0,087	0,623	0,461	**0,021**	0,812	**0,045**	**0,017**	**0,004**	0,203	0,960	0,239	0,797	0,576	0,079	0,051	0,138
**Adiponectin (μg/mL)**	**Correlation Coefficient**	-0,259	-0,197	-0,028	-0,071	**-0,276**	**-0,285**	0,079	-0,200	-0,182	**0,323**	-0,248	0,124	0,214	-0,043	0,096	-0,169
	***P***	0,054	0,141	0,838	0,596	**0,037**	**0,030**	0,554	0,133	0,170	**0,016**	0,061	0,361	0,111	0,751	0,469	0,204
**MPO (ng/mL)**	**Correlation Coefficient**	-0,123	0,163	0,097	0,113	**0,321**	-0,054	**0,264**	-0,022	-0,006	-0,030	**0,445**	-0,120	-0,159	0,197	-0,104	0,165
	***P***	0,352	0,214	0,449	0,377	**0,012**	0,676	**0,037**	0,863	0,963	0,819	**<0.01**	0,361	0,222	0,129	0,419	0,201
**oxLDL (U/l)**	**Correlation Coefficient**	**-0,285**	0,210	-0,148	0,062	0,058	-0,061	-0,102	0,057	0,031	0,153	**-0,312**	-0,067	0,140	-0,133	0,036	-0,200
	***P***	**0,018**	0,085	0,222	0,609	0,631	0,615	0,396	0,637	0,797	0,216	**0,009**	0,589	0,252	0,273	0,766	0,094

Correlation analysis was performed with the Pearson correlation test for all variables and with Spearman’s rank correlation test for leptin, Cd, Ba, and Tl. The level of significance was set as 0.05. Significant p are in bold. CRP, C-reactive protein; IL-6, interleukin-6; TNF-α, tumor necrosis factor-α; MPO, myeloperoxidase; oxLDL, oxidized low-density lipoprotein; V, Vanadium; Cr, Chromium; Mn, Manganese; Fe, Iron; Co, Cobalt; Ni, Nickel; Cu, Copper; Zn, Zinc; As, Arsenic; Se, Selenium; Rb, Rubidium; Sr, Strontium; Cd, Cadmium; Cs, Caesium; Ba, Barium; Tl, Thallium.

We further addressed the associations between the above statistically significant correlations. Linear regression models were applied with the following models: 1) crude model: no adjustment; 2) adjustment for age, gender, and BMI; 3) adjustment for age, gender, BMI, physical activity level, smoking, presence of any of the metabolic disorders (hypertension, hyperglycemia, and hyperlipidemia), and dietary intake of the respective metal (if available from Nutritionist Pro analysis). Only associations that showed a significant result in all three models are presented in **Table 4**. **V** was negatively associated with oxLDL (Beta^3^ ± SE= -0.014 ± 0.005, p=0.007), and Zn with leptin (Beta^3^ ± SE= -12.390 ± 5.226, p=0.025). Co associated negatively with adiponectin (Beta^3^ ± SE= -0.030 ± 0.012, p=0.014) and positively with MPO (Beta^3^ ± SE= 0.002 ± 0.001, p=0.023). Finally, Rb was negatively associated with oxLDL (Beta^3^ ± SE= -1.139 ± 0.411, p=0.008) and positively with MPO (Beta^3^ ± SE= 0.324 ± 0.102, p=0.003).

**Table 4:** Linear regression models of plasma metals in association with inflammatory/oxidative stress. biomarkers.

	Model 1^a^		Model 2^b^		Model 3^c^	
	Beta ± SE	*P*	Beta ± SE	*P*	Beta ± SE	*P*
V						
**oxLDL**	-0.010 ± 0.004	**0.018**	-0.010 ± 0.005	**0.034**	-0.014 ± 0.005	**0.007**
Co						
**Adiponectin**	-0.019 ± 0.004	**0.037**	-0.022 ± 0.009	**0.024**	-0.030 ± 0.012	**0.014**
**MPO**	0.002 ± 0.001	**0.012**	0.002 ± 0.001	**0.004**	0.002 ± 0.001	**0.023**
Zn						
**Leptin**	-4.184 ± 2.244	**0.067**	-6.266 ± 3.191	**0.054**	-12.390 ± 5.226	**0.025**
Rb						
**oxLDL**	-1.034 ± 0.384	**0.009**	-0.953 ± 0.365	**<0.01**	-1.139 ± 0.411	**0.008**
**MPO**	0.329 ± 0.086	**<0.01**	0.344 ± 0.086	**<0.01**	0.324 ± 0.102	**0.003**

a crude model,

b adjusted for age, gender, and BMI,

c adjusted for age, gender, BMI, physical activity level, smoking, presence of any of the metabolic disorders (hypertension, hyperglycemia, and hyperlipidemia), and dietary intake of the respective metal (if available from Nutritionist Pro analysis). Values resulted from linear regression models. The level of significance was set as 0.05. MPO, myeloperoxidase; oxLDL, oxidized low-density lipoprotein; V, Vanadium; Co, Cobalt; Zn, Zinc; Rb, Rubidium. Values in bold are significant (p<0.05).

## Discussion

Our study examined the relationship between plasma metals/metalloids and inflammatory/oxidative stress biomarkers in metabolically unhealthy Greek individuals with central obesity. For the first time, several associations among a number of metals, oxLDL, MPO, and the hormones leptin and adiponectin, are documented, supporting the role of metals in chronic inflammation and oxidative stress.

The association of circulating metals with the presence of obesity and its related comorbidities, as well as with anthropometric parameters has been well documented. In the National Health and Nutrition Examination Survey (NHANES) study, blood lead (Pb) exhibited a negative linear association with obesity, and high blood lead was associated with a lower risk of dyslipidemia only in obese participants (19). Data from the same study suggested that metals can even affect a high-quality diet since the association of the Healthy Eating Index-2015 (HEI-2015) score with central obesity was attenuated by higher levels of Pb, Cd, and Hg (12). In a US epidemiological investigation, Ba and Tl were positively associated with BMI and WC, whereas Cd, Co, Cs, and Pb were negatively correlated (20). Similarly, the Environmental Risk Score, a measure examining cumulative exposure to a mixture of metals, was associated with BMI, skinfold thickness, total body fat, hypertension, and type-2 diabetes mellitus (T2DM) (21).

Although metals and the risk of obesity have been the subject of extended epidemiological research, the precise mechanism underlying this association is still unknown. Since inflammation and oxidative stress are major contributors to the pathophysiology of obesity, and metals are implicated in both pathways, here, we address for the first time their associations that may partially explain the above mechanisms. In our study, Se positively correlated with TNF-a and adiponectin, Cu with CRP, leptin, and MPO, and negatively with TNF-a. Finally, Ni exhibited a negative correlation with adiponectin and leptin, and Fe with leptin. Cu, Se, Ni, and Fe levels have been associated with higher metabolic syndrome risk, as well as with the number of metabolic components and cardiometabolic parameters, such as higher BMI, glucose, and lower HDL (22–25). However, in our population, the above correlations did not remain significant when adjusting for potential confounders.

When adjusting for age, gender, BMI, physical activity level, smoking, and the presence of any of the metabolic disorders, V was negatively associated with oxLDL (Beta^3^ ± SE= -0.014 ± 0.005, p=0.007). According to recent research, V may have a protective role against obesity and metabolic disorders, partly through inhibiting phosphatases and affecting kinases that are essential for the insulin pathway (26). The majority of the literature supports an anti-obesity, hypolipidemic, antidiabetic, and antioxidant activity of V despite the contradictory findings, with studies showing that plasma V is related to higher dyslipidemia risk (27) and V supplementation in mice results in lipid accumulations in the hepatocytes (28). More specifically, obese people have 30% lower serum V levels than healthy controls, and serum V has an inverse association with BMI (4). Additionally, decreased body weight gain, lipids, and glucose levels as well as a suppression of preadipocyte differentiation and adipogenesis were observed in high-fat diet (HFD) mice given Jeju ground water containing V components compared to control mice (29). In a different study, a 4-week V supplementation reduced malondialdehyde (MDA) and inhibited endoplasmic reticulum stress and inflammatory response in addition to controlling body weight gain in HFD mice (30). In the majority of diabetes-induced animal studies, V administration was able to regulate oxidative stress biomarkers by restoring enzyme production at normal levels, such as raising catalase or glutathione peroxidase (31).

A negative association of Co with adiponectin (Beta^3^ ± SE= -0.030 ± 0.012, p=0.001) and a positive one with MPO (Beta^3^ ± SE= 0.002 ± 0.001, p=0.023) were calculated. There is conflicting information in the literature regarding the impact of Co on obesity and related comorbidities. Inorganic Co has been shown to possess a preventive role in obesity-related diseases by increasing leptin, adiponectin, and HDL and by regulating glucose and adipose cell size in HFD mice (32). On the contrary, in 47,595 women participating in the Sister Study, hypertension risk was associated with higher residential exposure to Co (33). In the NHANES cohort, urinary Co was negatively correlated with obesity in children/adolescents (34) and with HOMA-IR in female adults (35) in the NHANES cohort. Hu et al. (36) found that participants with higher urinary Co had a higher prevalence of obesity, insulin resistance, higher WC, and triglycerides, and lower HDL. According to a dose–response analysis, there is no evident safe threshold level below which Co exhibits no toxic effects. Paustenbach and his colleagues (37) suggested that lipid peroxidation and production of reactive oxygen species are two mechanisms maintaining Co toxicity. This may partly explain the positive association with MPO observed herewith. However, due to its capacity to cause hypoxia, Co is known to exhibit toxic effects in the cardiovascular, hematological, and neurological systems. Male Wistar rats orally exposed to Co chloride showed an increase in oxidative stress markers (H_2_O_2_ generation and MDA contents) and a concurrent decline in antioxidant enzymes such as catalase and glutathione peroxidase (38).

Regarding Zn, its plasma levels were negatively associated with leptin (Beta^3^ ± SE= -12.390 ± 5.226, p=0.025). Zn is known for its implication in several biochemical and metabolic processes, including energy metabolism and regulation of chronic inflammation and oxidative stress (39). Consequently, research primarily focusing on the control of lipids, insulin resistance, oxidative stress, and inflammation has simultaneously demonstrated its significant role in obesity and metabolic disorders (39, 40). Weight management, insulin resistance (41), and leptin regulation (42) have all been demonstrated as benefits of Zn supplementation in animal models, while clinical trials did not show such an impact (43). However, in line with our findings, plasma Zn was found to be adversely correlated with plasma leptin in 45 obese diabetic women (44), suggesting that Zn mediates the effects of leptin.

A negative association of Rb with oxLDL (Beta^3^ ± SE= -1.139 ± 0.411, p=0.008) and a positive one with MPO (Beta^3^ ± SE= 0.324 ± 0.102, p=0.003) were reported. Recently, Rb emerged as a new marker of obesity, detected at elevated concentrations in all tissues of high-caloric diet rats, and was strongly correlated with body weight gain and abdominal fat depots (45). However, according to relevant research, Rb exerts a protective impact against diabetes and a negative correlation with hypertension risk (46, 47). The role of Rb in oxidative stress is not fully explored. In endurance athletes, it was correlated with MDA (48), assuming that this relationship resulted from its capacity to exchange with K in chemical reactions of the energy production process (49, 50). More specifically, Rb may increase as a result of the resistance exercise-induced formation of reactive oxygen species (ROS) in order to facilitate the exchange of K into the cell (51). The contradictory results of our study, which showed a positive association of Rb with MPO and a negative one with oxLDL, may be partially explained by the lack of adjustment for dietary intake of Rb (not provided in nutrient analysis) or by other unpredictable confounders since the exact mechanism of Rb’s role in obesity has not yet been fully elucidated.

The present work constitutes the first attempt at investigating the potential links between environmental metals and obesity-related inflammation and oxidative stress. There are some limitations, i.e., the relatively small sample. Also, despite the adjustment of a large number of potential confounders, we cannot exclude the possibility of residual confounding, as in the case of Rb. On the contrary, we consider as a significant strength the application of ICP-MS, a high-throughput approach with extremely low detection limits simultaneously quantifying 16 metals, providing a full profile of the circulating metals. Finally, all the methodologies used herein are of great sensitivity and specificity.

## Conclusion

Overall, our results show that circulating V associates with oxLDL, Co with adiponectin and MPO, Zn with leptin, and Rb with MPO and oxLDL in individuals with obesity and metabolic disorders. These findings highlight the relationship of circulating metals with inflammatory and oxidative stress biomarkers and can be considered to be of importance given the impact of environmental factors on the development of obesity and metabolic abnormalities. Although the data obtained should be confirmed in larger studies, the outcomes of the present work enhance our knowledge of the pathophysiological role of environmental factors in obesity and metabolic-related disorders.

## Data availability statement

The raw data supporting the conclusions of this article will be made available by the authors, without undue reservation.

## Ethics statement

The studies involving humans were approved by Harokopio University Ethics Committee. The studies were conducted in accordance with the local legislation and institutional requirements. The participants provided their written informed consent to participate in this study.

## Author contributions

CA: Formal Analysis, Investigation, Writing – original draft. SK: Investigation, Methodology, Writing – review & editing. S-AK: Investigation, Writing – review & editing. DT: Investigation, Writing – review & editing. AS: Investigation, Writing – review & editing. EV: Investigation, Writing – review & editing. AG: Investigation, Writing – review & editing. NK: Methodology, Writing – review & editing. AK: Conceptualization, Methodology, Supervision, Writing – review & editing.
